# Electrocardiogram-gated Kilohertz Visualisation (EKV) Ultrasound Allows Assessment of Neonatal Cardiac Structural and Functional Maturation and Longitudinal Evaluation of Regeneration After Injury

**DOI:** 10.1016/j.ultrasmedbio.2019.09.012

**Published:** 2020-01

**Authors:** Raphael F.P. Castellan, Adrian Thomson, Carmel M. Moran, Gillian A. Gray

**Affiliations:** ⁎ Centre for Cardiovascular Science, Queen's Medical Research Institute, The University of Edinburgh, Edinburgh, UK; † Edinburgh Imaging, Queen's Medical Research Institute, The University of Edinburgh, Edinburgh, UK

**Keywords:** Echocardiography, Ultrasound, Cardiac Regeneration, Cardiac Physiology

## Abstract

The small size and high heart rate of the neonatal mouse heart makes structural and functional characterisation particularly challenging. Here, we describe application of electrocardiogram-gated kilohertz visualisation (EKV) ultrasound imaging with high spatio-temporal resolution to non-invasively characterise the post-natal mouse heart during normal growth and regeneration after injury. The 2-D images of the left ventricle (LV) acquired across the cardiac cycle from post-natal day 1 (P1) to P42 revealed significant changes in LV mass from P8 that coincided with a switch from hyperplastic to hypertrophic growth and correlated with *ex vivo* LV weight. Remodelling of the LV was indicated between P8 and P21 when LV mass and cardiomyocyte size increased with no accompanying change in LV wall thickness. Whereas Doppler imaging showed the expected switch from LV filling driven by atrial contraction to filling by LV relaxation during post-natal week 1, systolic function was retained at the same level from P1 to P42. EKV ultrasound imaging also revealed loss of systolic function after induction of myocardial infarction at P1 and regain of function associated with regeneration of the myocardium by P21. EKV ultrasound imaging thus offers a rapid and convenient method for routine non-invasive characterisation of the neonatal mouse heart.

## Introduction

The heart undergoes significant changes during the early period after birth that are important for subsequent function and susceptibility to disease (Soonpaa et al. 1996; Soonpaa and Field 1998; Leu et al. 2001; Hirschy et al. 2006; Corrigan et al. 2010; Piquereau et al. 2010; Haubner et al. 2012; Porrello et al. 2013; Tian et al. 2014; Blom et al. 2016). Furthermore, retention of regenerative capacity in neonatal mouse and human hearts after injury such as myocardial infarction (MI) (Haubner et al. 2012; Porrello et al. 2013; Porrello and Olson 2014; Haubner et al. 2016; Castellan and Meloni 2018) has made this early post-natal period a key focus for investigation. Whereas embryonic cardiac organogenesis has been extensively studied in the mouse (Vincent and Buckingham 2010), much less is known of the functional and structural changes during early post-natal maturation of the mammalian heart or the potential for non-invasive *in vivo* imaging to reflect these changes and the impact of genetic interventions on them.

Magnetic resonance imaging can provide high-resolution 3-D images of mouse hearts from as early as postnatal day 0 (P0) (Petiet et al. 2008), and this approach has been used for basic extrapolation of left ventricular mass and function in neonatal mice (Gunadasa-Rohling et al. 2018). However, the challenge of maintaining physiologic viability during relatively long scanning times of 35 min to 3 h, availability of a suitable scanner and high costs relating to this technique limit its routine use for neonatal characterisation. Cardiac computerised tomography, has been applied in neonatal mice to detect congenital heart defects (Kim et al. 2013), but its relatively low resolution and the requirement for injection of contrast agent has limited its application mostly to adult animals (Clark and Badea 2014). Ultrasound has been extensively used in the context of cardiovascular research both in the mouse embryo and the adult (Phoon and Turnbull 2003; Corrigan et al. 2010; Moran et al. 2013). The strengths of this technique are its high resolution (50 µm), its rapidity (10–15 min scans per animal) and its relatively low cost (Olive and Tuveson 2006). However, its poor spatiotemporal resolution in neonatal mice, because of their small size and high heart rate, had previously restricted its use to Doppler and 1-D measurements in the context of normal cardiac growth and only the latter during regeneration after injury (Zhou et al. 2003; Haubner et al. 2012; Corrigan et al. 2013;Porrello et al. 2013; Blom et al. 2016; Reynolds et al. 2016; Das et al. 2019). High-frame-rate electrocardiogram-gated kilohertz visualisation (EKV) imaging enables post-acquisition generation of a single typical cardiac cycle. This is generated from many sequentially acquired M-modes that are spatially and temporally interleaved into an ultrasound B-mode image data set (Moran et al. 2013). EKV imaging is known to increase spatial resolution of cardiac borders, as well as temporal resolution of the systolic and diastolic ends of the cardiac cycle (Lindsey et al. 2018), but its use has not previously been reported in neonatal mice.

The primary aim of the present study was therefore to test the potential for EKV ultrasound imaging to detect structural changes during hyperplastic and hypertrophic post-natal heart growth and to identify contemporaneous changes in systolic and diastolic function. EKV imaging was also applied for detection of cardiac regeneration after neonatal myocardial infarction.

## Materials and Methods

### Ethics statement

All experiments were approved by and performed in accordance with the University of Edinburgh Animal Welfare and Ethical Review Body and the UK Home Office.

### Animals

All animals were C57 BL/6 J mice bred in house from stock originally obtained from Harlan (Bicetser, Oxfordshire, UK) and aged post-natal day 1 (P1) to P42. Males were used for the normal growth study and both males and females for the neonatal cardiac injury study. Mice were kept under a 12-h light/12-h dark cycle mimicking circadian rhythm. Temperature of the environment was maintained at 21 ± 2°C and humidity at 50 ± 10%. Animals had access to chow diet and water *ad libitum.*

### EKV ultrasound imaging procedure

**Fig. 1:**
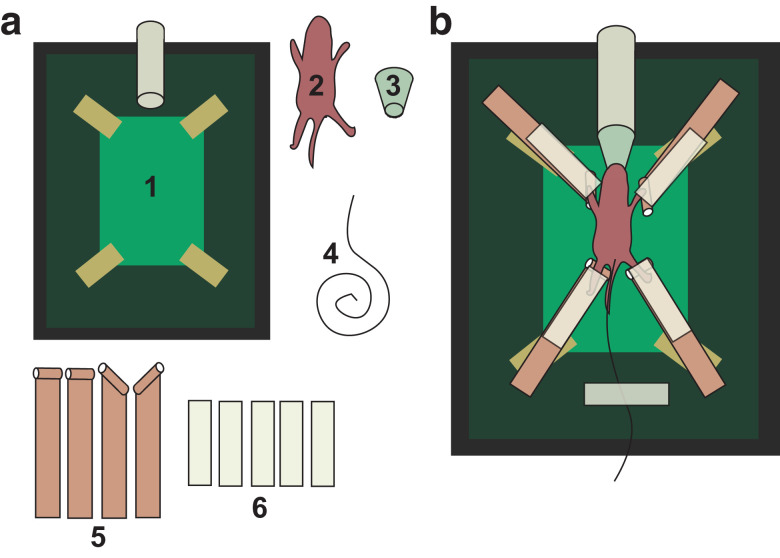
Setup for assessing neonatal cardiac function and structure using high-resolution *in vivo* ultrasound imaging. (a) Adult setup for ultrasound imaging. A standard ultrasound table (*1*) was used. The neonatal pup (*2*) was placed on the table, which was adapted using a nose cone and adapter (*3*), a neonatal rectal thermometer (*4*), conductive copper tape (*5*) and regular tape (*6*) as shown on panel b.

Anaesthesia was induced using isoflurane (Merial Animal Health Ltd, Bracknell, Berkshire, UK) in medical O_2_ (BOC Medical, Manchester, UK) at concentrations: 5% (P2, P4, P8) and 4%–5% (P21, P42). Mice were placed in a supine position on a heated ultrasound table (40°C). Adapters were used to deliver anaesthesia throughout the procedure for adequate effective delivery of anaesthesia (Fig. 1a, 1b). The diameters of the adapters used were Ø5 mm (P2 and P4) and Ø8 mm (P8). A standard nose cone was used for mice aged P21 and P42. Isoflurane was used at the following concentrations: 3%–4% (P2, P4, P8) and 2%–3% (P21, P42) for anaesthesia maintenance. Rectal temperature was monitored (36.5–38°C) with a Physitemp RET-4 and RET-3 (Physitemp, Clifton, NJ, USA) for neonates and juvenile/adult mice respectively. Of note, although pedal reflex is absent in neonatal mice under typical anaesthesia concentration (∼2%), we found that insertion of a neonatal mouse rectal thermometer still elicited reflexes at this level of anaesthesia. We therefore decided to increase the concentration of anaesthesia, resulting in the use of 4% in the younger pups. Thoracic hair was removed where necessary using commercially available electric shavers and depilatory cream. Conductive copper tape (Direct Products, Clitheroe, Lancashire, UK) was used to extend the ultrasound table's existing electrodes (Fig. 1a, 1b) and allow recording of the electrocardiogram in neonatal mice. The copper tape was wrapped around a cotton bud's stalk to avoid excessive bending of the pup's limbs. Conductive ultrasound gel (Spectra 360 Electrode Gel, Parker Laboratories Inc., Fairfield, NJ, USA) was then applied to each paw to record electrocardiogram signals. Pre-warmed Aquasonic 100 ultrasound transmission gel (Parker Laboratories Inc.) was applied onto the abdomen avoiding bubble formation.

### Ultrasound Traces and Analysis

Image acquisition was performed on a VisualSonics Vevo® 770 ultrasound biomicroscope (Visualsonics, Amsterdam, North Holland, The Netherlands). The probes used were: RMV704 for pups aged P2, P4 and P8 (Centre frequency: 40 MHz, axial resolution: 40 μm, focal length: 6 mm) and RMV707 B for animals aged P21 and P42 (centre frequency: 30 MHz, axial resolution: 55 μm, focal length: 12.7 mm). Cardiac structure and function were assessed using standard parasternal long axis (PLAX) images of the left ventricle (LV) in B-mode and M-mode. Functional measurements of the heart were acquired using mid-ventricular pulse wave Doppler from an apical 4-chamber view of the heart with the Doppler sample volume placed within the LV cavity below the aortic and mitral valves and aligned with the aortic valve. From this view, the ratio of the blood velocities at E (early peak diastolic flow) to A (late peak diastolic flow) was used to determine diastolic function and myocardial performance index (MPI) for global ventricular function (Zhou et al. 2003; Corrigan et al. 2010) (Fig. S1). For B-mode imaging, the probe was placed to achieve a PLAX view of the LV and EKV imaging of the heart was performed (Moran et al. 2013). Using this technique, images are acquired over multiple cardiac cycles and are post-processed using an image reconstruction process to produce a movie representing a single typical heart cycle containing many more frames of ultrasound data than could be acquired in real time. This method allowed identification of the LV endocardial and epicardial boundaries at the end of systole and diastole, which were used by the Vevo 770 software to calculate structural (LV mass, wall thickness, left ventricle end-systolic area [LVESA] and left ventricle end-diastolic area [LVEDA]) and functional (fractional area change [FAC] and ejection fraction [EF]) parameters (Fig. S2). From the PLAX B-mode view the M-mode cursor was placed to span across the left ventricular anterior wall, mid-cavity and posterior wall, allowing visualisation of wall movement along a single line. Those traces were used to determine end diastolic and systolic distances, which were used by the Vevo 770 software to calculate fractional shortening (Fig. S3).

Representative videos 1–4 of each imaging mode can be found online.

### Tissue collection and processing

Fresh heart samples were washed in saline (phosphate-buffered saline, Merck, Gillingham, UK) then fixed in 10% formalin (Merck) for 24 h and subsequently placed in 70% ethanol (Merck) for at least 24 h before embedding in paraffin (Merck). Paraffin-embedded samples were cut into sequential 4 µm thick sections on superfrost slides (VWR, Lutterworth, UK). Blood samples were obtained from neonatal mice after decapitation and plasma isolated through centrifugation for later analysis.

### Histologic and immunofluorescence staining

Slides were deparaffinised and rehydrated. Histologic analysis was performed on haematoxylin (Pioneer Research Chemicals Limited, Colchester, UK) and eosin (ThermoFisher Scientific, Basingstoke, UK) stained sections. For immunofluorescent staining, antigen retrieval was carried out in boiling sodium citrate buffer (10 mM, pH6, Merck, Gillingham, UK). Non-specific staining was blocked with 10% normal goat serum (Biosera, Heathfield, UK) and stained with isolectin B4 (IB4, 1/100, ThermoFisher Scientific) followed by streptavidin coupled Alexa Fluor 488 (1/200, ThermoFisher Scientific) and Cyanin3 coupled anti–α smooth muscle actin (αSMA, 1/75, Merck) to identify endothelial and mural cells, or IB4 (1/100) followed by streptavidin coupled Alexa Fluor 488 (1/200) and rhodamine-coupled wheat germ agglutinin (WGA, 1/75, Vector Laboratories Ltd, Peterborough, UK) to identify cardiomyocyte membranes and measure cardiomyocyte cross-sectional area (CMCSA). To identify proliferative cardiomyocytes, cell membranes were permeabilised (0.2% triton x-100) and slides incubated in blocking solution of 20% normal goat serum and 5% bovine serum albumin (Merck) in tris buffer saline (Merck), and then stained with mouse anti-cardiac troponin T (cTnT, 1/100, Abcam, Cambridge, UK) and rabbit anti-Ki67 (1/200, Abcam) in blocking solution with 0.1% Tween (Merck) followed by goat anti-mouse Alexa Fluor 488 (1/200, ThermoFisher Scientific) and goat anti-rabbit Alexa Fluor 555 (1/200, ThermoFisher Scientific). Nuclei were stained with 1/1000 4′,6 diamino-2-phenylindole (DAPI, Merck).

### Image analysis

Pictures were taken using a Zeiss Axiovert200 microscope or a Zeiss Axio Scan.Z1 slide scanner at magnification × 40 (Zeiss, Oberkochen, Germany). For measurement of CMCSA, four fields of view within the outer myocardium were obtained because this is where the cardiomyocytes lay perpendicular to the short axis view. Ten CMCSA were measured in each field of view. For measurement of cardiomyocyte proliferation, vascular density and vessel diameter, five regions only containing myocardium were drawn around and their areas measured. Counting of the number of Ki67^+ve^ nuclei within cTnT^+ve^ cardiomyocytes was performed within each region and normalised to its area. For vascular density, each vessel was counted and its diameter measured within each area. The number of αSMA^+ve^ vessels was counted within the whole left ventricle. Analysis was performed using Adobe Photoshop CS6 (Adobe Inc., San Jose, CA, USA).

### Induction of MI in neonatal mice

Myocardial infarction was induced by ligation of the left anterior descending (LAD) coronary artery in P1 mice as described by Haubner et al. (2012) and Porrello et al. (2013). Briefly, anaesthesia was induced by a combination of hypothermia and breathable (isoflurane) anaesthetic. The animal was secured onto an ice pack for the duration of the procedure to maintain adequate anaesthesia. A 5 mm skin incision was placed over the left thorax above the fifth rib, the skin was blunt dissected and a small opening was created at the fifth intercostal space to visualise the left pulmonary lobes and heart. The LAD coronary artery was identified and ligated (9-0 Ethilon, Ethicon, Somerville, New Jersey, USA). The ribs, pectoral muscles and skin were closed (8-0 Prolene, Ethicon). Analgesia was administrated with one drop of 1/50 bupivacaine (Marcain Polyamp Steripack 0.25%, AstraZeneca, Cambridge, UK) in 0.9% saline (B Braun, Melsungen, Germany) onto the wound. The entire litter was tattooed for identification purposes, dabbed with material from the maternal cage and returned to the nest. Studies were conducted at day 1 and day 21 post-MI to respectively assess successful induction of injury and regeneration with ultrasound, histologic and biochemical techniques.

### Cardiac troponin assay

Cardiac troponin I (cTnI) levels were measured in plasma samples obtained at day 1 after MI as an indicator of initial injury using a high sensitivity kit (Life Diagnostics, West Chester, PA, USA) following manufacturer's instructions.

### Statistical analysis

Statistical analysis was performed using GraphPad Prism 5 software (GraphPad Software Inc., San Diego, CA, USA). Data are expressed as mean ± standard error of the mean. Student's unpaired *t*-test was used for comparison of two groups. Comparison between more than two groups used a one-way analysis of variance with *post hoc* Bonferroni multi-comparison test, as indicated in the text. Significance level was set to *p* < 0.05.

## Results

### EKV high-resolution ultrasound imaging can be used to image the neonatal mouse heart

**Fig. 2:**
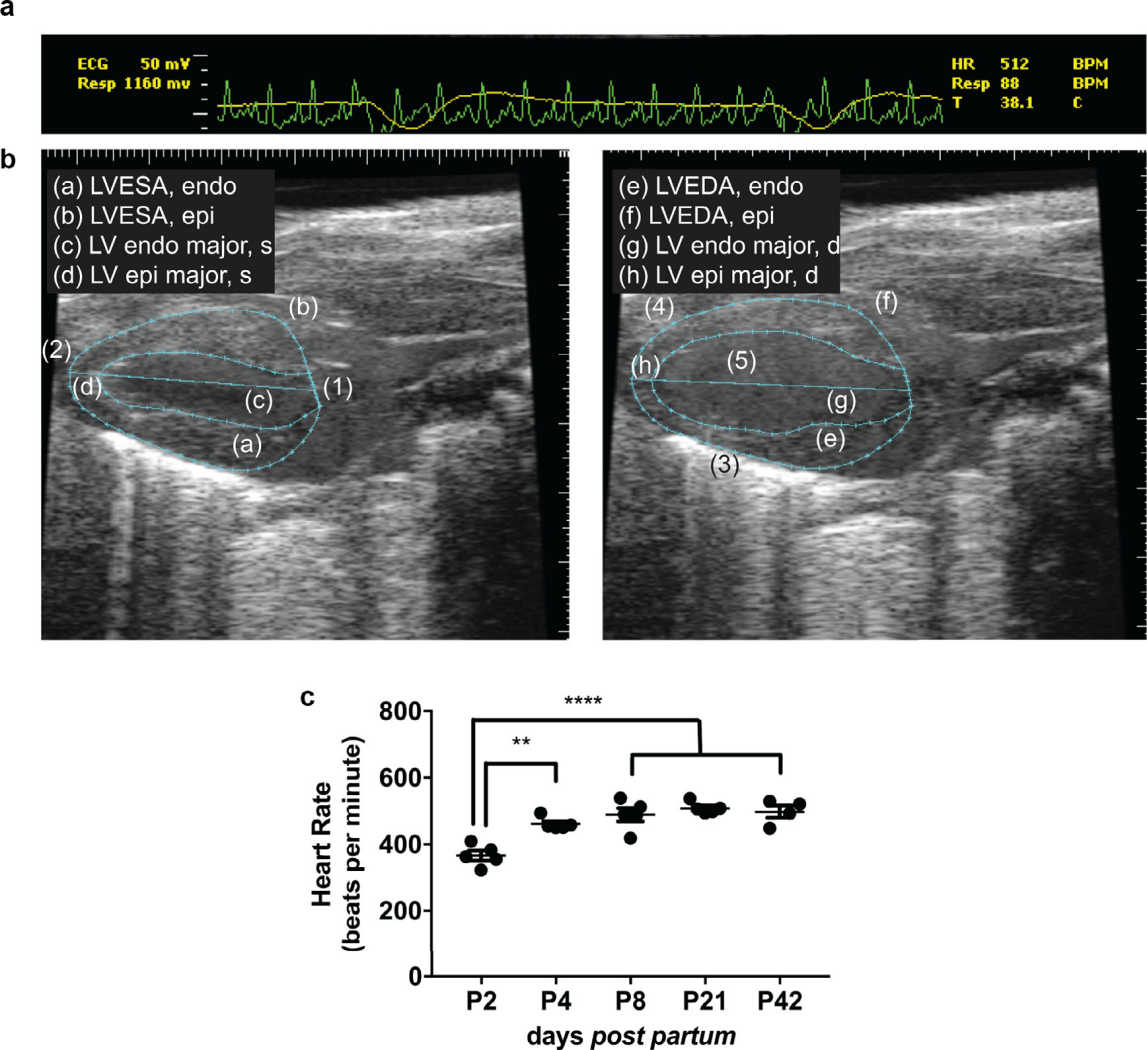
PLAX EKV B-mode imaging in neonatal mice. The adapted ultrasound setup allowed recording of electrocardiogram, respiration and temperature (*a*) and thereby to perform electrocardiogram-gated kilohertz visualisation (EKV) imaging on parasternal long axis (PLAX) B-mode imaging (*b*). (*a*) and (*b*) show representative traces of a mouse on post-natal day 2. The Vevo770 software was used to identify left ventricular end-systolic and end-diastolic areas of the endocardium (LVESA, endo [a], and LVEDA, endo [e]) and epicardium (LVESA, epi [b], and LVEDA, epi [f]), LV endocardial and epicardial majors at the end of systole (LV endo major, s [c] and LV epi major, s [d]) and at the end of diastole (LV endo major, d [g], and LV epi major, d [h]). Anatomic features are (1) aortic valve, (2) apex, (3) LV posterior wall, (4) LV anterior wall, (5) LV cavity. Heart rate was evaluated from this imaging modality (*c*).

As shown in Figure 2, electrocardiograms were successfully detected in neonatal mice allowing acquisition of EKV ultrasound traces (Fig. 2a, 2b) and reduced variability of LV border detection compared with standard B-mode (Fig. S4). Heart rate could also be obtained (Fig. 2c) and was maintained within a physiologic range during the procedure for all ages of mice.

### LV mass obtained through EKV high-resolution ultrasound imaging correlates with gravimetric LV mass during early post-natal growth

**Fig. 3:**
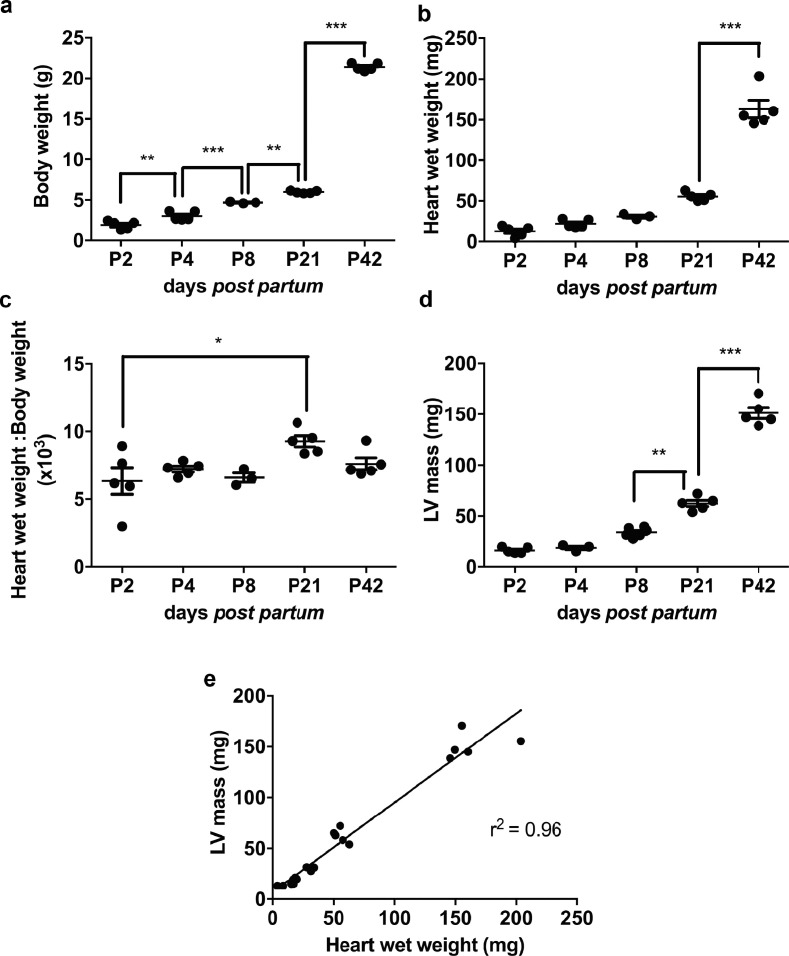
Measurements extracted with electrocardiogram-gated kilohertz visualisation (EKV) ultrasound imaging correlate with *ex vivo* measurements. Body weight (a) was measured before high-resolution *in vivo* ultrasound. Heart wet weight (b) was measured immediately after high-resolution *in vivo* ultrasound imaging after dissection and used to calculate heart to weight ratio (c). EKV B-mode allowed calculation of left ventricular (LV) mass (d). LV mass was plotted against heart wet weight for each animal and linear regression applied (e) to obtain the coefficient of determination *r*^2^. All measurements were performed at post-natal day 2 (P2), P4, P8, P21 and P42; n = 3–5/group. Results are presented as mean ± standard error of the mean; *p* value determined with one-way analysis of variance with Bonferroni *post hoc* test. *p < 0.05, **p < 0.01, ***p < 0.001.

To investigate whether information obtained from EKV ultrasound imaging on cardiac structures correlated with *ex vivo* measurements during early life, cardiac growth from P2 to P42 was studied *in vivo* by ultrasound and compared with *ex vivo* metrics. As shown in Figure 3a, body weight significantly increased between each time point (*p* < 0.0001). *Ex vivo* heart wet weight tended to increase from P2 but was only increased significantly between P21 and P42 (*p* < 0.0001; Fig. 3b). The heart wet weight to body weight ratio was constant, indicating parallel growth, with the exception of P21, when heart growth exceeded that of the body (*p* < 0.05; Fig. 3c). LV mass obtained from EKV imaging in the long axis largely reflected the pattern of change in *ex vivo* heart wet weight, with significant increase in weight from P8 onward (P8–P21 *p* < 0.01, P21–P42 *p* < 0.001; Fig. 3d) and indeed the two measures were strongly correlated (Fig. 3e).

### The increase in LV mass detectable by high-resolution ultrasound correlates temporally with a shift from cardiomyocyte hyperplasia to hypertrophy and with maturation of the vasculature

**Fig. 4:**
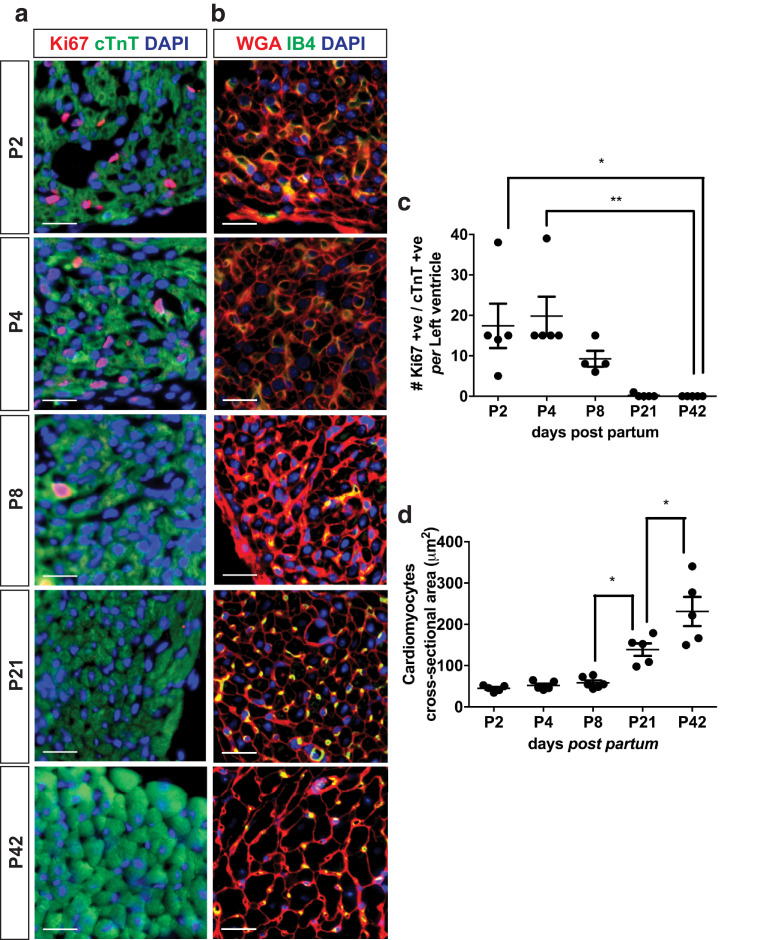
Distinct phases of growth of the post-natal mouse heart. Proliferative cardiomyocytes were identified as positive for anti-cardiac troponin T (cTnT; green-cardiomyocytes marker) and containing a nucleus, 4′,6 diamino-2-phenylindole (DAPI; blue), positive for Ki67 (red-proliferation maker) (a). Double-positive nuclei appear pink. Quantification was performed on five different field of views across the whole left ventricle of 4 μm thick cardiac sections. Cardiomyocyte cross-sectional area (CMCSA) (b) was investigated on wheat germ agglutinin (WGA; red-membrane marker), isolectin B4 (IB4; green-endothelial cells marker) and DAPI (blue) stained 4 μm thick sections. Vessels appear yellow as double positive for IB4 and WGA. Quantification of CMCSA was performed on five different fields of view within the outer layer of the left ventricle where cardiomyocytes are perpendicular to the plan of section. Scale bars = 20 μm. (c) Cardiomyocyte proliferation quantification; (d) CMCSA quantification. All measurements were performed at post-natal day 2 (P2), P4, P8, P21 and P42; n = 3–5/group. Results are presented as mean ± standard error of the mean; *p* value determined with one-way analysis of variance with Bonferroni *post hoc* test. *p < 0.05, ***p < 0.001.

Cardiomyocyte proliferation dominated during the first day of post-natal growth as indicated by a high density of cTnT^+ve^, Ki67^+ve^, DAPI^+ve^ cells (Fig. 4a, 4c). This did not, however, result in a significant change in heart mass that could be detected by either gravimetric or ultrasound analysis (Fig. 3). In contrast, the shift from hyperplastic to hypertrophic growth, reflected in increasing CMCSA (Fig. 4b, 4d) from post-natal day 8 (*p* < 0.05), correlated with the timing of increased ultrasound-derived LV mass. The density of proliferative cardiomyocytes decreased rapidly to practically non-detectable levels during this period (Fig. 4a, 4c).

**Fig. 5:**
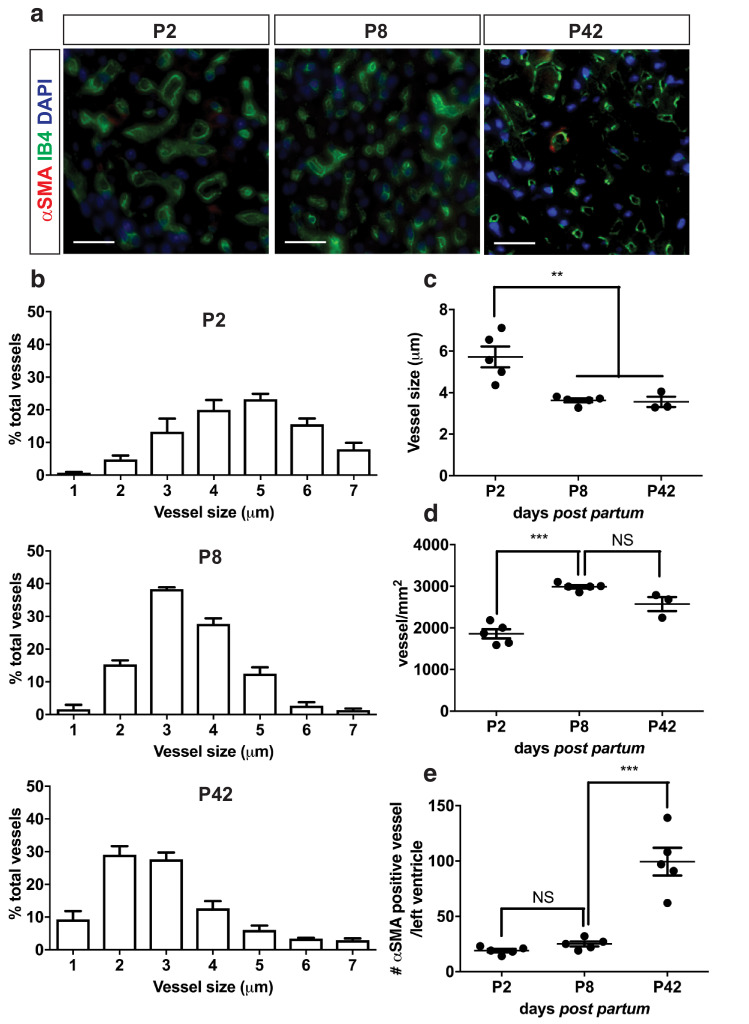
Vessel size, density and maturation in the post-natal mouse heart. Vessel size, density and maturation were investigated on isolectin B4 (IB4; green-endothelial cells), α-smooth muscle actin (αSMA; red-mural cell coated vessels) and 4′,6 diamino-2-phenylindole (DAPI; blue). Quantification of vessel size and density was performed on five different field of views within the outer layer of the left ventricle on 4 μm thick stained sections at post-natal day 2 (P2), P8 and P42. (a) Representative images; scale bars = 50 μm. (b) Vessel size distribution was investigated at the three chosen time points. (c) Average vessel size in micrometres. (d) Vessel density was expressed per square millimetre of myocardium. (e) Number of αSMA^+ve^ vessels within the whole left ventricle; n = 3–5/group. Results are presented as mean ± standard error of the mean; *p* value determined with one-way analysis of variance with Bonferroni *post hoc* test; *p* > 0.05 (nonsignificant), **p < 0.01, ***p < 0.001.

The increase in LV mass and CMCSA was accompanied by changes in the pattern of vascularisation of the LV (Fig. 5a), such that a reduction in average vessel size (Fig. 5b, 5c) was associated with an increase in vessel density as the heart matured (Fig. 5d). An increase in the number of αSMA-positive vessels was identified between ventricles of neonatal (P2 and P8) compared with young adult mice (P42, *p* < 0.001, Fig. 5e) consistent with vessel maturation during this period.

### Structural measurements extracted from EKV ultrasound imaging identifies distinct phases of post-natal cardiac structural and functional maturation

**Fig. 6:**
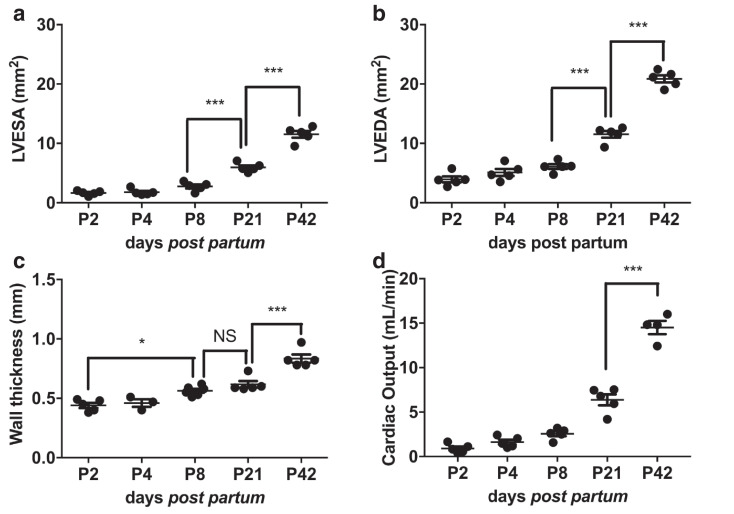
EKV ultrasound imaging allows investigation of cardiac structural changes *in vivo* from early neonatal to adult stages. Electrocardiogram-gated kilohertz visualisation (EKV) B-mode allowed calculation of (a) left ventricle end-systolic area (LVESA), (b) left ventricle end-diastolic area (LVEDA), (c) left ventricle wall thickness and (d) cardiac output; n = 3–5/group. Results are presented as mean ± standard error of the mean; *p* value determined with one-way analysis of variance with Bonferroni *post hoc* test. *p* > 0.05 (nonsignificant), *p < 0.05, ***p < 0.001.

The area of the ventricle at the end of systole (LVESA) and diastole (LVEDA) were both relatively stable until P4 but increased significantly from P8 onward (*p* < 0.001; Fig. 6a, 6b), consistent with other assessments of heart growth during this period. Wall thickness increased significantly between each time point except between P8 and P21, when the increase appeared interrupted (Figs. 4d, 6c). Expanding ventricular dimensions resulted in an increased capacity for cardiac output (Fig. 6d).

### Systolic functional capacity remains stable throughout post-natal and early adult life

**Fig. 7:**
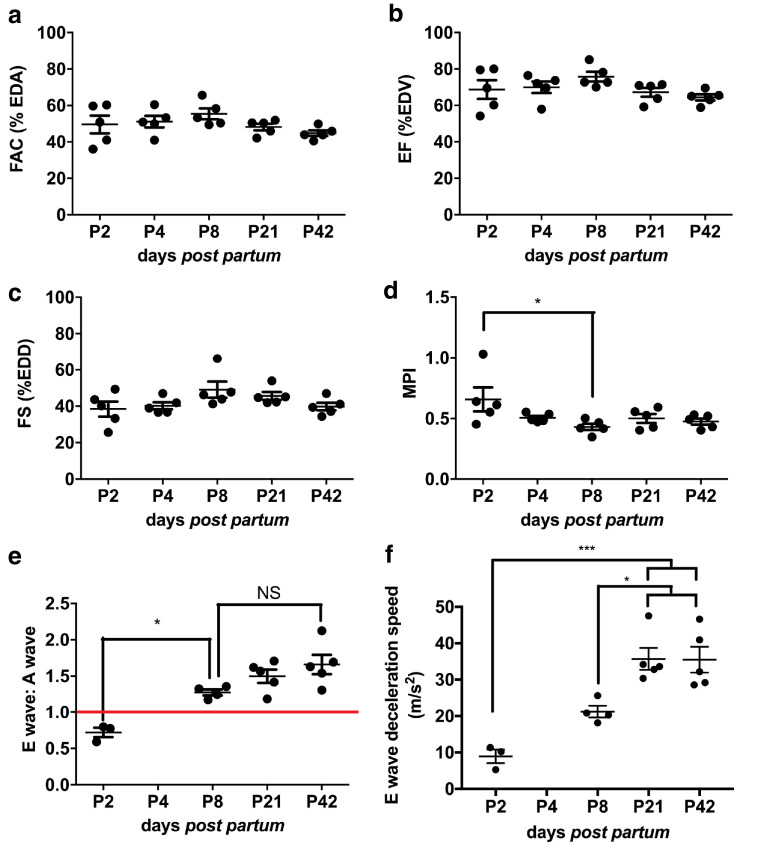
Cardiac systolic function is stable throughout postnatal cardiac structural changes. Cardiac systolic function was investigated using high-resolution *in vivo* electrocardiogram-gated kilohertz visualisation (EKV) ultrasound imaging at post-natal day 2 (P2), P4, P8, P21 and P42. EKV B-mode allowed calculation of (a) fractional area change (FAC) and (b) ejection fraction (EF). (c) Parasternal long axis (PLAX) M-mode was used to obtain fractional shortening (FS). Mid-ventricular pulse wave Doppler waveforms enabled calculation of the myocardial performance index (MPI) (d) as well as the diastolic function parameter of E to A wave ratio (e) and E wave deceleration (f); n = 3–5/group. Results are presented as mean ± standard error of the mean. *p*-value determined with one-way analysis of variance with Bonferroni *post hoc* test. *p < 0.05, ***p < 0.001.

Although ventricular area was increasing during post-natal life, it is interesting to note that relative functional capacity was maintained during this period, whether measured from a long axis view in B-mode (FAC, Fig. 7a; EF, Fig. 7b) or in M-mode (fractional shortening, Fig. 7c).

MPI collected from the mid-ventricular Doppler trace indicated a marginal increase in global ventricular performance at P8 compared with P2 (*p* < 0.05). It was, however, unchanged between all other timepoints. The switch from atrial contraction (A wave in the mitral valve Doppler trace; Fig. S1d) to ventricular relaxation (E wave in the mitral valve Doppler trace; Fig. S1d) in driving left ventricular filling during diastole that occurs during the first day of neonatal life (Zhou et al. 2003), was also detected in the neonatal mouse heart. Thus, the E/A wave ratio (Fig. 7e) switched from <1 to >1 in the neonatal period between P2 and P8 (*p* < 0.05) and remained at >1 thereafter. E wave deceleration (Fig. 7f), another readout of ventricular diastolic function, increased significantly from P2.

### EKV high-resolution ultrasound imaging is a valuable tool for functional and structural analyses during neonatal cardiac regeneration

**Fig. 8:**
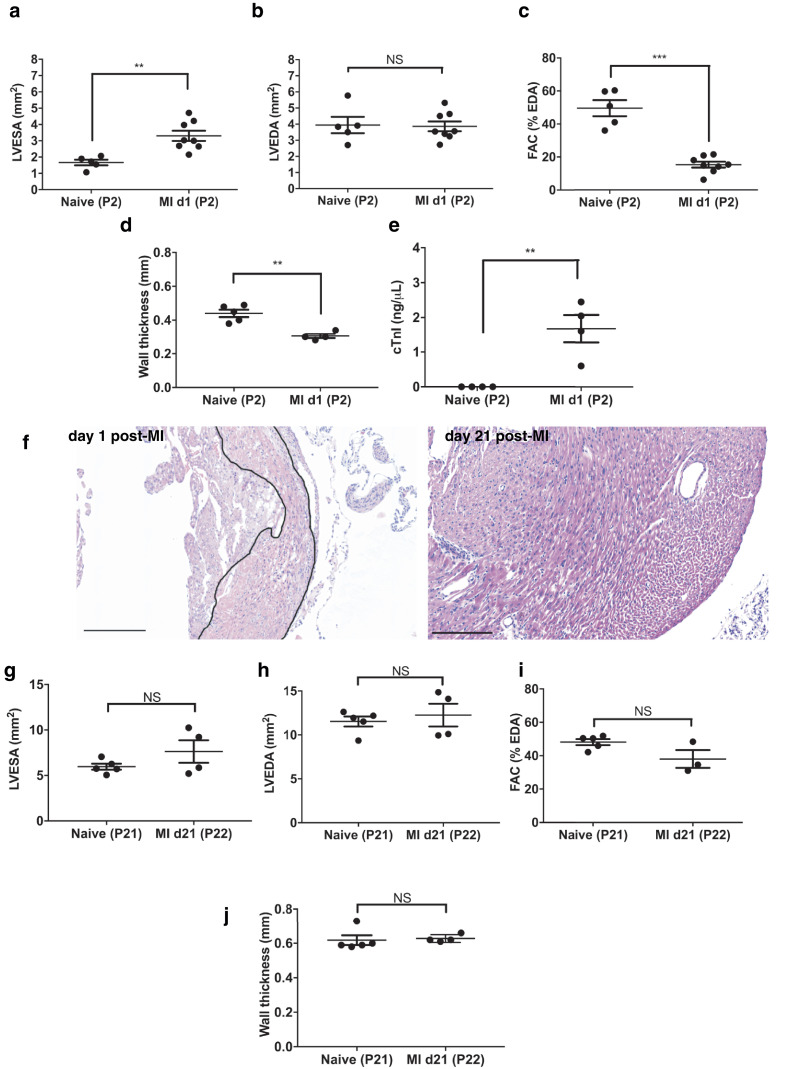
High-resolution EKV imaging *in vivo* ultrasound can be used to confirm presence of injury and regeneration after LAD ligation in P1 mice. Mice underwent left anterior descending (LAD) coronary artery ligation at post-natal day 1 (P1). Cardiac function was assessed with electrocardiogram-gated kilohertz visualisation (EKV) ultrasound imaging. This was used to calculate left ventricle end-systolic area (LVESA), left ventricle end-diastolic area (LVEDA) and fractional area change (FAC) at day 1 (a–c) and day 21 post–myocardial infarction (MI) (g, h, i). Functional decrease was used to characterise successful LAD ligation at day 1. Functional recovery was also noted at day 21 as previously described in the literature (Haubner et al. 2012; Porrello et al. 2013). Functional decrease and recovery were respectively associated with signs of injury (*black outline*) and normal morphology (f, *left* and *right panel,* respectively) within the left ventricle on haematoxylin-eosin–stained 4 μm thick cardiac sections. Wall thickness was calculated at day 1 (d) and day 21 (j) post-MI and compared with naïve animals. Plasma cardiac troponin I (cTnI) levels were measured and indicated increased levels at day 1 after LAD ligation compared with naïve animals, indicative of cardiac injury (e); n = 3–8/group. Results are presented as mean ± standard error of the mean. ***p < 0.001, Student's *t*-test. Scale bars = 200 μm.

After confirmation of the utility of non-invasive EKV ultrasound imaging to assess normal ventricular growth in the early neonatal period, we next sought to determine whether it could be applied to confirm cardiac injury and regeneration in the neonatal mouse. Myocardial infarction was induced in P1 mice by permanent occlusion of the LAD coronary artery as described earlier. Traces acquired from EKV ultrasound imaging at P2 revealed a significant increase of LVESA (Fig. 8a), accompanied by retention of LVEDA (Fig. 8b), resulting in a reduction in FAC (Fig. 8c), reflecting loss of systolic function. Successful induction of injury was confirmed by terminating a subset of animals at this stage. cTnI concentration was significantly increased in the plasma (Fig. 8e), and signs of myocardial injury were evident on histologic sections of heart (Fig. 8f, left panel). By day 21 post-MI both LVESA and LVEDA were similar to those of naïve animals (Fig. 8g, 8h), which translated to recovery of FAC compared with P2 imaging (Fig. 8i). This was associated with normal wall thickness (Fig. 8j) and morphology (Fig. 8f, right panel), indicative of regeneration of the myocardium after injury at P1.

Representative videos 5–6 can be found on the online version.

## Discussion

High-resolution *in vivo* ultrasound is a widely used and powerful tool to study structure and function in the adult mouse heart, including changes after MI (Moran et al. 2013), as well as functional maturation of the mouse embryonic heart (Phoon and Turnbull 2003; Yu et al. 2008; Corrigan et al. 2010). Its application in neonatal mice has, however, thus far been restricted to Doppler and 1-D measurements during normal growth (Zhou et al. 2003; Corrigan et al. 2013; Reynolds et al. 2016) and solely the latter after MI and during regeneration (Haubner et al. 2012; Porrello et al. 2013; Das et al. 2019). Here, we optimised a setup to permit *in vivo* cardiac ultrasound in neonatal mice that allowed gating on the electrocardiogram signal and thereby acquisition of 2-D high-resolution movies of the LV across an entire cardiac cycle using EKV. By doing so, we were able to resolve LV boundaries more accurately than with standard B-mode. We could then extract extensive *in vivo* structural information and for the first time correlate with known functional maturation of the mouse neonatal heart during normal growth, as well as identifying structural and functional changes associated with neonatal cardiac injury and regeneration.

In the first postnatal week mouse cardiac growth is mostly achieved through hyperplasia (Soonpaa et al. 1996; Leu et al. 2001). As expected, early hyperplastic growth was characterised by a high density of Ki67^+ve^ cardiomyocytes within the myocardium up to P8, whereas CMCSA remained stable. Interestingly, whereas neither *ex vivo* heart weight nor ultrasound detected LV mass or internal dimensions changed during this period, wall thickness obtained through EKV *in vivo* imaging did significantly increase, providing evidence for tissue expansion driven by cardiomyocyte proliferation (Foglia and Poss 2016). As the heart grows, it is supplied by an extensive vascular network that, until birth, remains mostly segregated to the outer, or compact, myocardium. This was denominated by Tian et al. (2014) as the first coronary vascular population (CVP). The inner part of the ventricular myocardium, the trabeculated myocardium, is mostly supplied in oxygen and nutrients through diffusion from the ventricular cavities. After birth, compaction of most of the trabeculated myocardium is accompanied by the formation of a second CVP proposed to originate from the endocardium (Tian et al. 2014). The timeline leading to the formation of a mature capillary network in the postnatal heart, however, remained unclear. Here, we identified an increasing vessel density during the first postnatal wk that was accompanied by a reduction of vessel size within the compact myocardium, indicative of branching.

The initial hyperplastic phase is followed by a switch to hypertrophic growth, which was confirmed here by a significant decrease in the density of Ki67 positive cardiomyocytes and increased cross-sectional area between P8, P21 and P42 agreeing with previously published data (Soonpaa et al. 1996; Leu et al. 2001). Non-invasive imaging permitted detection of an increase in LV mass from P8 onward, coinciding with the switch to hypertrophic growth. Of note, despite increased LV mass as well as increased end-systolic and end-diastolic dimensions of the LV, wall thickness was unchanged between P8 and P21, indicative of structural reorganisation of the myocardium. Interestingly, Piquereau et al. (2010) previously reported improved delivery of adenosine tri-phosphate to myofibrils within the cardiomyocytes during this phase, consistent with functional maturation. Recently, Sereti et al. (2018) examined cardiomyocyte clonal expansion in the growing heart and reported a decrease in the number of large clone clusters from P15. They proposed that this may be due to the migration of daughter cells past this point. Together with our *in vivo* and *ex vivo* data, this would suggest a phase of cardiomyocyte re-organisation in the juvenile mouse heart. Between P21 and P42, all parameters measured (such as increased CMCSA, LV mass, *ex vivo* heart weight, *etc.*) point to classic hypertrophic growth.

LV systolic function was maintained, despite the considerable maturational changes within the LV, during the entire period studied. In contrast, diastolic function, detected by mid-ventricular Doppler waveforms, changed dramatically between P2 and P8, moving from a foetal pattern of LV filling predominantly through atrial contraction (A wave > E wave), to an adult pattern of LV filling predominantly as a result of ventricular relaxation (A wave < E wave), consistent with previous observations (Zhou et al. 2003).

Cardiac regeneration after injury at P1 in the neonatal mice is a widely used model to identify regenerative mechanisms that might be applied in the adult (Haubner et al. 2012; Mahmoud et al. 2013; Porrello et al. 2013; Xin et al. 2013; Aurora et al. 2014;Lam and Sadek 2018). Non-invasive confirmation of initial injury is essential in these studies. Reports that utilise ultrasound as a way to confirm injury are solely based on 1-D M-mode imaging (Haubner et al. 2012; Porrello et al. 2013; Blom et al. 2016; Das et al. 2019). Here we show that EKV high-resolution ultrasound imaging permits detection of early structural and functional changes at day 1 post-MI. LV end systolic dimensions obtained with EKV imaging were increased after injury, with no change in diastolic dimensions, indicating contractile deficiency. This was also previously suggested by Haubner et al. (2012) based on 1-D M-mode measurements. Contractile deficiency was accompanied by a reduction in wall thickness that correlated with evidence of tissue injury on tissue sections, confirmed by increased circulating cTnI levels in trunk blood. Restoration of function and histologic evidence of structural integrity by day 21 post-MI were consistent with regeneration, as previously described in this model (Haubner et al. 2012; Porrello et al. 2013).

In summary, using EKV ultrasound imaging in neonatal mice we were able to collect *in vivo* information on structural and contemporaneous functional maturation from as early as P2. This enabled non-invasive detection of cardiac maturation in post-natal and juvenile mice that correlated with *in vitro* observations, as well as providing a means to detect functional changes associated with cardiac injury and regeneration. EKV high-resolution ultrasound imaging thus offers a practical 2-D *in vivo* tool to study the impact of genetic or other modification on the processes of neonatal cardiac growth and regeneration after injury.

## Appendix Supplementary materials

Fig. S1. Representative neonatal and adult mid-ventricular pulse wave Doppler traces and measurements obtained. Pulse wave Doppler traces were obtained from four-chamber apical view of the left ventricle by placing the cursor within the left ventricular (LV) cavity under mitral and aortic valves (c). (a) A typical neonatal (post-natal day 1 [P1]) Doppler trace with A > E wave. (b) A typical adult (P42) Doppler trace with A < E wave. (d) Schematic representation of an adult mid-ventricular Doppler trace with designation of the peak E wave velocity (E), peak A wave velocity (A), isovolumic contraction time (IVCT), ejection time (ET) and isovolumic relaxation time (IVRT). (e) Measurements details and formulas. Image, application 1

Fig. S2. Representative PLAX EKV B-mode still images and measurements obtained. Parasternal long axis (PLAX) view of the left ventricle (LV) was imaged throughout a cardiac cycle using electrocardiogram-gated kilohertz visualisation (EKV) B-mode ultrasound. Representative images of a 1-d-old mouse left ventricle (LV) at the end of systole (a) and diastole (b). The Vevo770 software was used to identify left ventricular end systole and diastole areas of the endocardium (LVESA, endo (a) and LVEDA, endo (e)) and epicardium (LVESA, epi (b) & LVEDA, epi (f)), LV endocardial and epicardial majors at the end of systole (LV endo major, s (c) & LV epi major, s (d)) and at the end of diastole (LV endo major, d (g) & LV epi major, d (h)). Anatomic features are (1) aortic valve, (2) apex, (3) LV posterior wall, (4) LV anterior wall, (5) LV cavity. (c) Measurements details and formulas. Image, application 2

Fig. S3. Representative M-mode still images and measurements obtained. Parasternal long axis (PLAX) view of the left ventricle (LV) was used to place the M-mode cursor (green line) spanning across the left ventricular anterior wall, cavity and posterior wall (a). Posterior and anterior endocardial wall movement along the cursor was traced as indicated by the blue lines on (b) across three cardiac cycles. LV end-diastolic and end-systolic diameters (EDD and ESD) are used to calculate the fractional shortening (FS). Those are representative images of a 1-d-old mouse LV. (c) Measurements details and formulas. Image, application 3

Fig. S4. Improved resolution of ventricular boundaries with electrocardiogram-gated kilohertz visualisation (EKV) imaging compared with conventional B-mode. (a) Still frames of neonatal mouse hearts at post-natal day 2 (P2) imaged in the parasternal long axis view with conventional B-mode (left) and EKV (right) indicate improved resolution of ventricular borders. Variance (b) and standard deviation (c) of measurements of left ventricular end-systolic area (LVESA) and end-diastolic area (LVEDA) indicate a clear reduction when obtained using EKV imaging compared with B-mode imaging; n = 5/group; *p* value determined with paired Student's *t*-test. * *p* < 0.05. Image, application 4

**Video 1: PLAX EKV B-mode (P2 mouse).** Typical parasternal long axis (PLAX) electrocardiogram gated kilohertz visualisation (EKV) B-mode trace of a mouse pup aged 2 d post-natal (P2).

**Video 2: PLAX M-mode (P2 mouse).** Typical parasternal long axis (PLAX) M-mode trace of a mouse pup aged 2 d post-natal (P2).

**Video 3: Doppler P2.** Typical mid-ventricular Doppler traces of a mouse pup aged 2 d post-natal (P2).

**Video 4: Doppler P8.** Typical mid-ventricular Doppler traces of a mouse pup aged 8 d post-natal (P8).

**Video 5: MI day 1.** Typical parasternal long axis (PLAX) electrocardiogram-gated kilohertz visualisation (EKV) B-mode trace of a mouse pup at day 1 after induction of myocardial infarction (MI) at post-natal day 1 (P1).

**Video 6: MI day 21.** Typical parasternal long axis (PLAX) electrocardiogram-gated kilohertz visualisation (EKV) B-mode trace of a mouse pup at day 21 after induction of myocardial infarction (MI) at post-natal day 1 (P1).
