# Supplementary material for: Electrocardiogram-gated Kilohertz Visualisation (EKV) Ultrasound Allows Assessment of Neonatal Cardiac Structural and Functional Maturation and Longitudinal Evaluation of Regeneration After Injury
Source: Ultrasound Med Biol. 2020 Jan;46(1):167–79. doi: 10.1016/j.ultrasmedbio.2019.09.012 (PMC6900752; doi:10.1016/j.ultrasmedbio.2019.09.012)
Supplement: Supplementary file 1 — Fig. S1. Representative neonatal and adult mid-ventricular pulse wave Doppler traces and measurements obtained. Pulse wave Doppler traces were obtained from four-chamber apical view of the left ventricle by placing the cursor within the left ventricular (LV) cavity under mitral and aortic valves (c). (a) A typical neonatal (post-natal day 1 [P1]) Doppler trace with A > E wave. (b) A typical adult (P42) Doppler trace with A < E wave. (d) Schematic representation of an adult mid-ventricular Doppler trace with designation of the peak E wave velocity (E), peak A wave velocity (A), isovolumic contraction time (IVCT), ejection time (ET) and isovolumic relaxation time (IVRT). (e) Measurements details and formulas. [file mmc1.pdf]

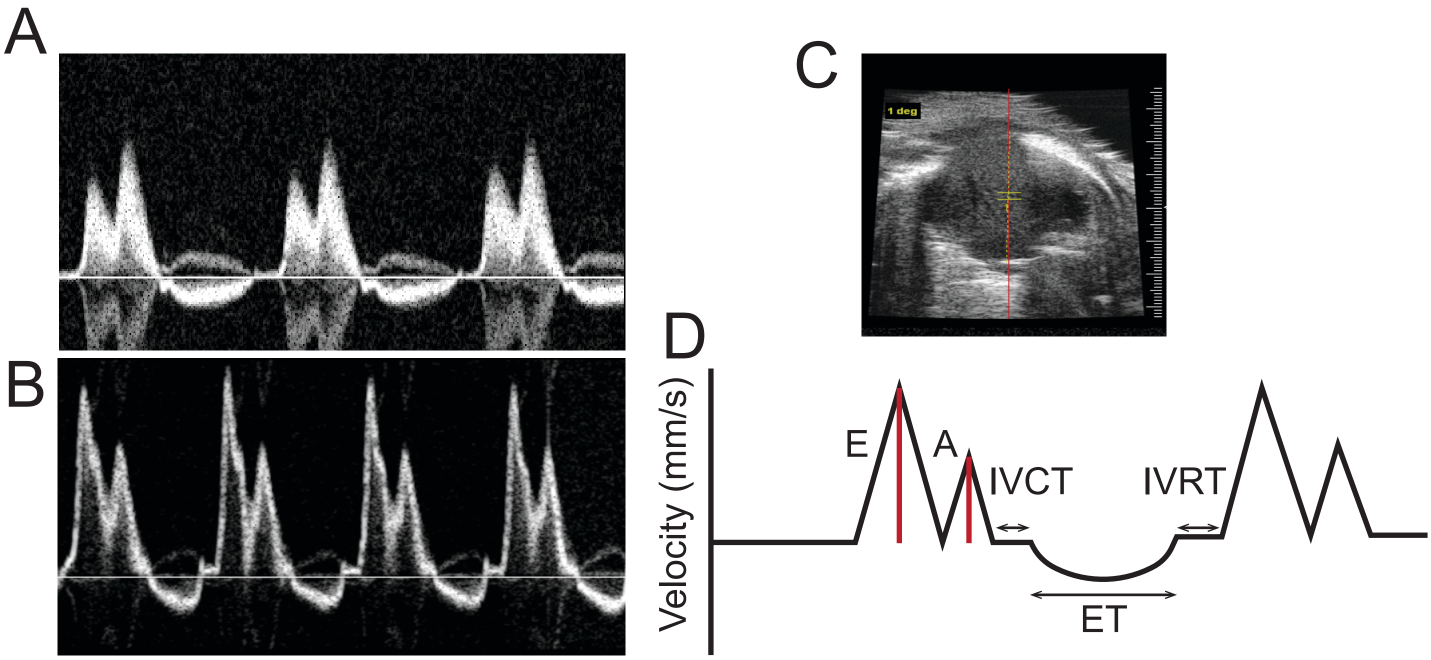

E

Parameter Short Name	Parameter Long Name	Formula	Units
IVCT (LV)	Left Ventricle Isovolumic Contraction Time	N/A	ms
IVRT (LV)	Left Ventricle Isovolumic Relaxation Time	N/A	ms
ET (LV)	Left Ventricle Ejection Time	N/A	ms
MV E	Mitral Valve Peak E Velocity	N/A	mm/s
MV A	Mitral Valve Peak A Velocity	N/A	mm/s
MPI	Myocardial Performance Index	$MPI = {(IVCT\ (LV) + IVRT(LV))}/{ET\ LV}$	N/A
E/A ratio	Mitral Valve E to A wave ration	$E/A\ ratio = {MV\ E}/{MV\ A}$	N/A
