# Supplementary material for: Electrocardiogram-gated Kilohertz Visualisation (EKV) Ultrasound Allows Assessment of Neonatal Cardiac Structural and Functional Maturation and Longitudinal Evaluation of Regeneration After Injury
Source: Ultrasound Med Biol. 2020 Jan;46(1):167–79. doi: 10.1016/j.ultrasmedbio.2019.09.012 (PMC6900752; doi:10.1016/j.ultrasmedbio.2019.09.012)
Supplement: Supplementary file 2 — Fig. S2. Representative PLAX EKV B-mode still images and measurements obtained. Parasternal long axis (PLAX) view of the left ventricle (LV) was imaged throughout a cardiac cycle using electrocardiogram-gated kilohertz visualisation (EKV) B-mode ultrasound. Representative images of a 1-d-old mouse left ventricle (LV) at the end of systole (a) and diastole (b). The Vevo770 software was used to identify left ventricular end systole and diastole areas of the endocardium (LVESA, endo (a) and LVEDA, endo (e)) and epicardium (LVESA, epi (b) & LVEDA, epi (f)), LV endocardial and epicardial majors at the end of systole (LV endo major, s (c) & LV epi major, s (d)) and at the end of diastole (LV endo major, d (g) & LV epi major, d (h)). Anatomic features are (1) aortic valve, (2) apex, (3) LV posterior wall, (4) LV anterior wall, (5) LV cavity. (c) Measurements details and formulas. [file mmc2.pdf]

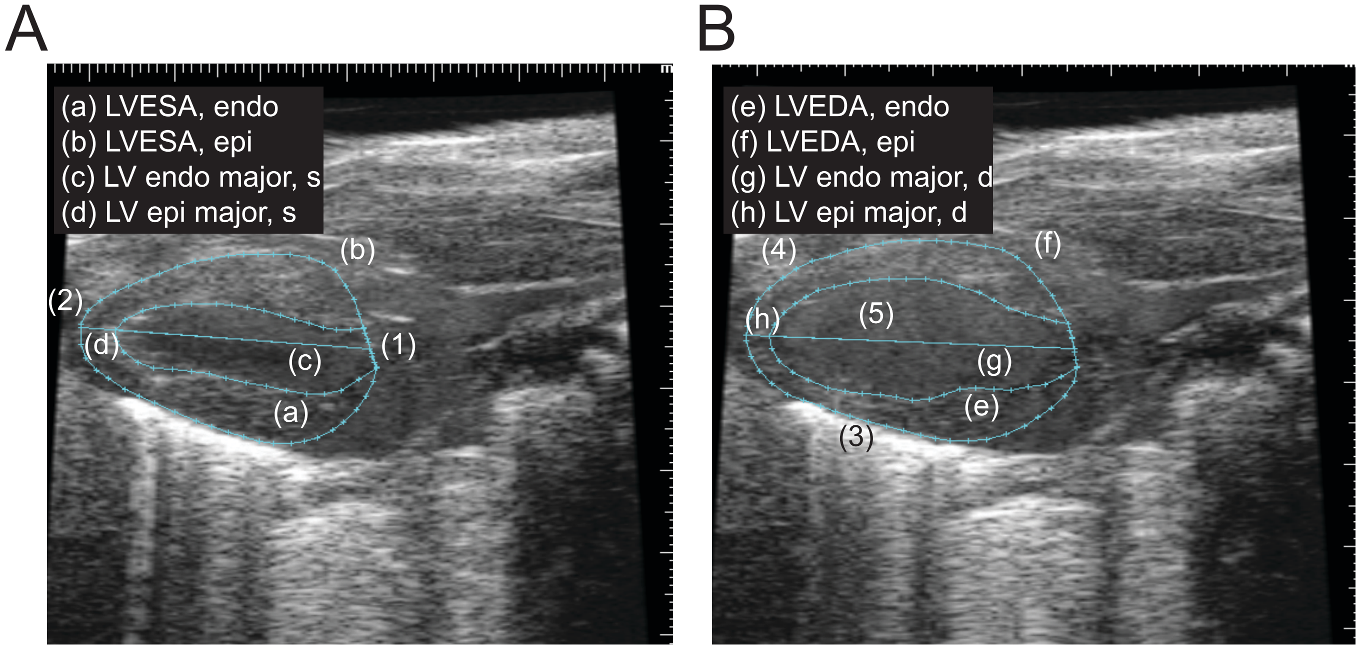

C

Parameter Short Name	Parameter Long Name	Formula	Units
LVESA, epi	Epicardial Left Ventricular End Systole Area	N/A	mm^2
LVESA, endo	Endocardial Left Ventricular End Systole Area	N/A	mm^2
LVEDA, epi	Epicardial Left Ventricular End Diastole Area	N/A	mm^2
LVEDA, endo	Endocardial Left Ventricular End Diastole Area	N/A	mm^2
LV Epi major, s	Left Ventricle Epicardial Major, End Systole	N/A	mm
LV Epi major, d	Left Ventricle Epicardial Major, End Diastole	N/A	mm
LV Endo major, s	Left Ventricle Endocardial Major, End Systole	N/A	mm
LV Endo major, d	Left Ventricle Endocardial Major, End Diastole	N/A	mm
LV Vol, d	Left Ventricle Volume, End Diastole	$LV\ Vol,d = \left(\frac{4\pi}{3}\right) x \left(\frac{LV\ endo\ major,d}{2}\right) x \left(\frac{LVEDA,endo}{\pi\left(\frac{LV\ endo\ major,d}{2}\right)}\right)^2$	µL
LV Vol, s	Left Ventricle Volume, End Systole	$LV\ Vol,s = \left(\frac{4\pi}{3}\right) x \left(\frac{LV\ endo\ major,s}{2}\right) x \left(\frac{LVESA,endo}{\pi\left(\frac{LV\ endo\ major,s}{2}\right)}\right)^2$	µL
LV SV	Left Ventricle Stroke Volume	$LV\ Vol,d - LV\ Vol,s$	µL
FAC	Fractional Area Change	$FAC = \left(\frac{(LVEDA,endo - LVESA,endo)}{LVEDA,endo}\right) x\ 100$	%
EF	Ejection Fraction	$EF = \left(\frac{LV\ SV}{LV\ Vol,d}\right) x\ 100$	%
WT	Average Wall Thickness	$WT = \sqrt{\frac{LVEDA,epi}{\pi}} - \sqrt{\frac{LVEDA,endo}{\pi}}$	mm
LV mass	Left Ventricle Mass	$LV\ mass = 1.05\ x \left(\left(\frac{5}{6}\right) x\ LVEDA,epi\ x\ (LV\ epi\ major,d + WT) - \left(\frac{5}{6}\right) x\ LVEDA,endo\ x\ LV\ endo\ major,d\right)$	mg
