# Supplementary material for: Electrocardiogram-gated Kilohertz Visualisation (EKV) Ultrasound Allows Assessment of Neonatal Cardiac Structural and Functional Maturation and Longitudinal Evaluation of Regeneration After Injury
Source: Ultrasound Med Biol. 2020 Jan;46(1):167–79. doi: 10.1016/j.ultrasmedbio.2019.09.012 (PMC6900752; doi:10.1016/j.ultrasmedbio.2019.09.012)
Supplement: Supplementary file 3 — Fig. S3. Representative M-mode still images and measurements obtained. Parasternal long axis (PLAX) view of the left ventricle (LV) was used to place the M-mode cursor (green line) spanning across the left ventricular anterior wall, cavity and posterior wall (a). Posterior and anterior endocardial wall movement along the cursor was traced as indicated by the blue lines on (b) across three cardiac cycles. LV end-diastolic and end-systolic diameters (EDD and ESD) are used to calculate the fractional shortening (FS). Those are representative images of a 1-d-old mouse LV. (c) Measurements details and formulas. [file mmc3.pdf]

A

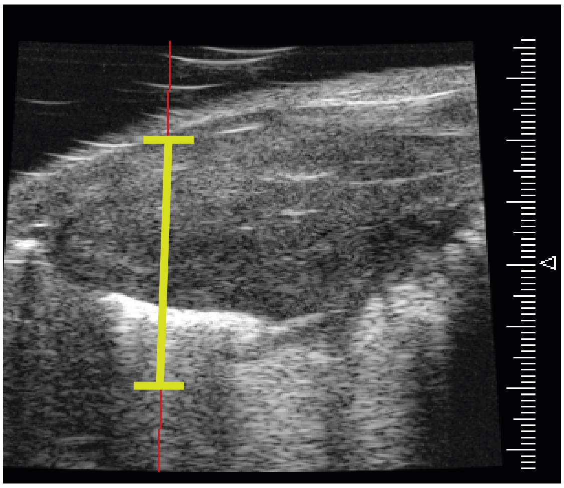

B

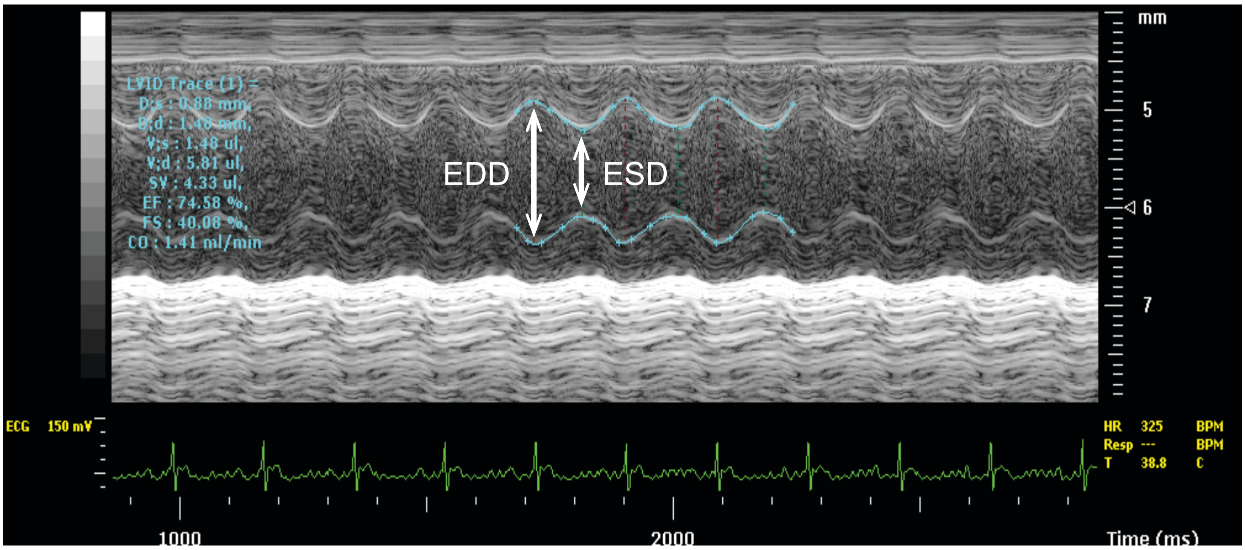

C

Parameter Short Name	Parameter Long Name	Formula	Units
ESD	Left Ventricle End Systole Distance	N/A	mm
EDD	Left Ventricle End Diastole Distance	N/A	mm
FS	Fractional Shortening	$FS = \left({(EDD - ESD)}/{EDD} \right) x\ 100$	%
