# Supplementary figures and images for: Electrocardiogram-gated Kilohertz Visualisation (EKV) Ultrasound Allows Assessment of Neonatal Cardiac Structural and Functional Maturation and Longitudinal Evaluation of Regeneration After Injury
Source: Ultrasound Med Biol. 2020 Jan;46(1):167–79. doi: 10.1016/j.ultrasmedbio.2019.09.012 (PMC6900752; doi:10.1016/j.ultrasmedbio.2019.09.012)

A

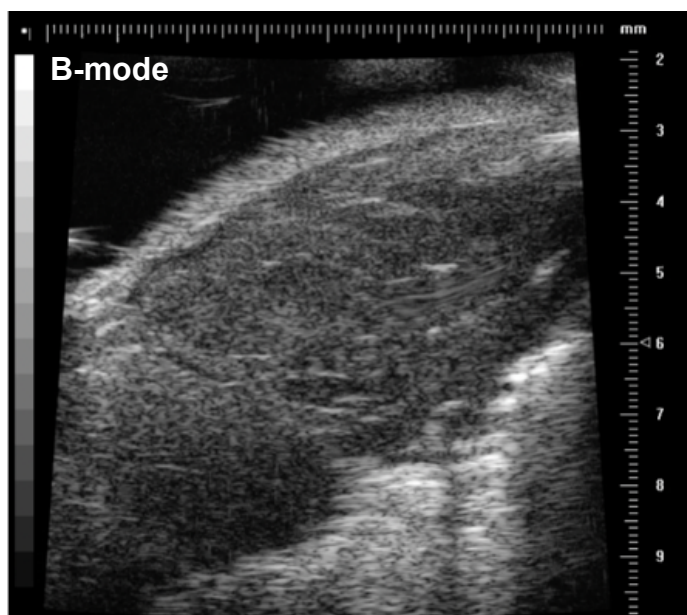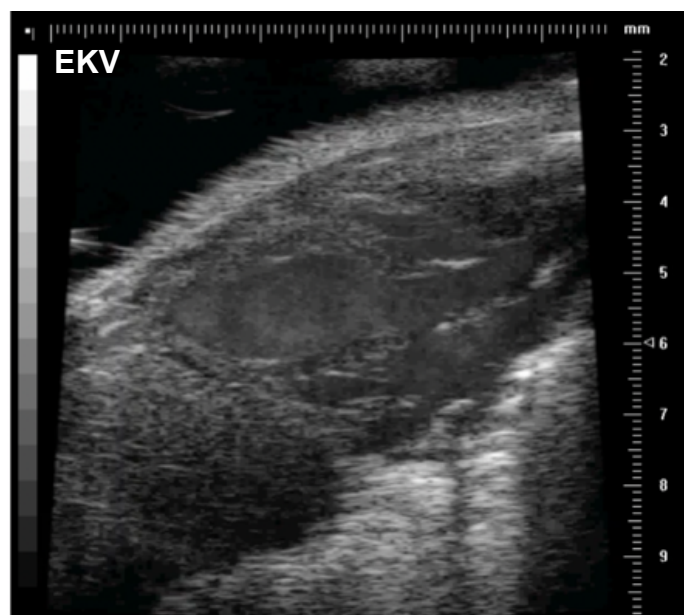

B

LVESA

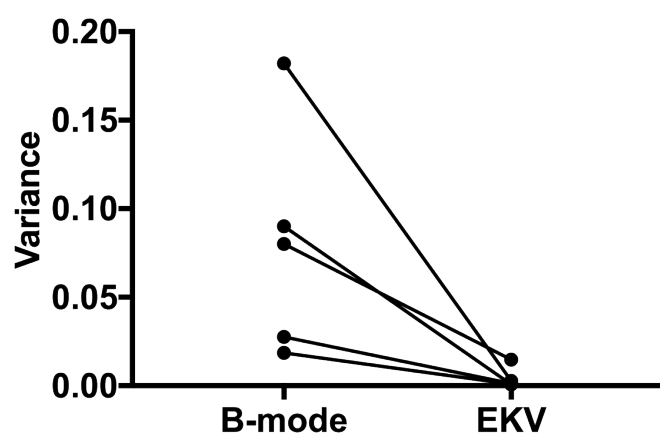

LVEDA

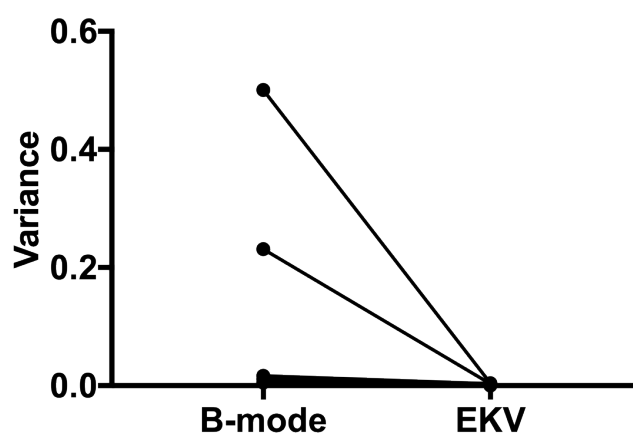

C

LVESA

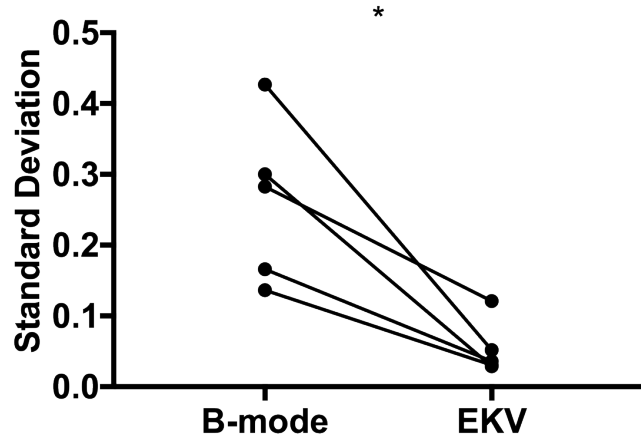

LVEDA

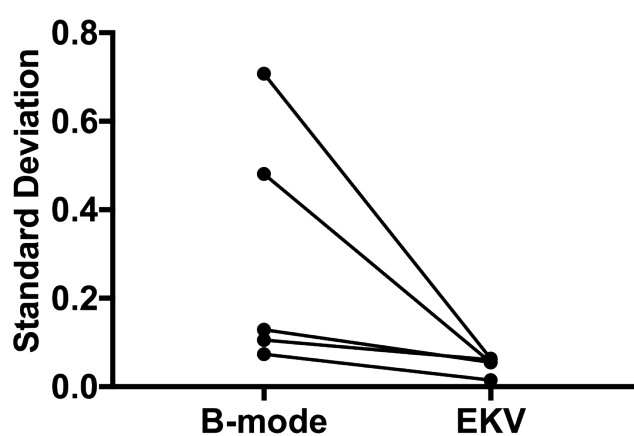

Supplement: Supplementary file 4 — Fig. S4. Improved resolution of ventricular boundaries with electrocardiogram-gated kilohertz visualisation (EKV) imaging compared with conventional B-mode. (a) Still frames of neonatal mouse hearts at post-natal day 2 (P2) imaged in the parasternal long axis view with conventional B-mode (left) and EKV (right) indicate improved resolution of ventricular borders. Variance (b) and standard deviation (c) of measurements of left ventricular end-systolic area (LVESA) and end-diastolic area (LVEDA) indicate a clear reduction when obtained using EKV imaging compared with B-mode imaging; n = 5/group; p value determined with paired Student's t-test. * p < 0.05. [file mmc4.pdf]
